# Supplementary material for: Mammalian SWI/SNF chromatin remodeler is essential for reductional meiosis in males
Source: Nat Commun. 2021 Nov 12;12:6581. doi: 10.1038/s41467-021-26828-1 (PMC8589837; doi:10.1038/s41467-021-26828-1)
Supplement: Supplementary file 1 — Supplementary Information [file 41467_2021_26828_MOESM1_ESM.pdf]

Supplemental Information

Mammalian SWI/SNF chromatin remodeler is essential for reductional meiosis in males

Debashish U. Menon[1], Oleksandr Kirsanov[2], Christopher B. Geyer[2,3] & Terry Magnuson[1]

Supplemental Figures 1-10

Supplementary tables 1-5

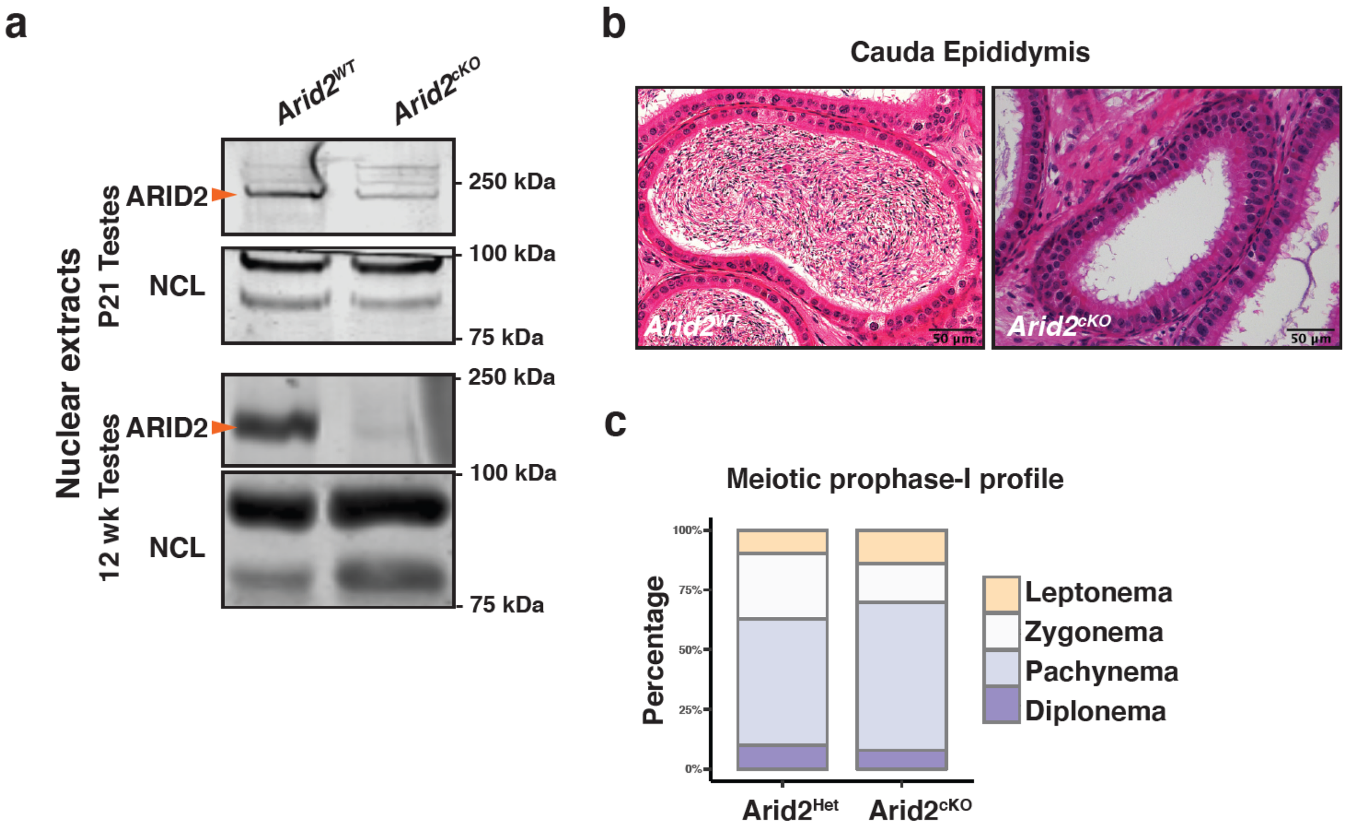

Supplementary Figure 1. Characterization of ARID2 during spermatogenesis. (a) ARID2 immunoblot on nuclear extracts from *Arid2*^{WT} and *Arid2*^{cKO} spermatogenic cells obtained from juvenile (P21), adult (12-week) testes. Nucleolin (NCL) is a nuclear loading control. Target proteins are labeled with orange arrowhead. Immunoblots were repeated twice for juvenile and thrice for adult samples with similar results. (b) H&E-stained paraffin sections of cauda epididymides obtained from adult *Arid2*^{WT} *and Arid2*^{cKO} mice. Scale bar: 50 µm, magnification: 25x. Images are representative of histology obtained from three separate *Arid2*^{WT} *and Arid2*^{cKO} epididymal sections. (c) Meiotic prophase-I profiles determined from juvenile *Arid2*^{Het} *and Arid2*^{cKO} spermatocyte spreads. Prophase-I substages were identified by SYCP3 and γH2Ax immunostaining.

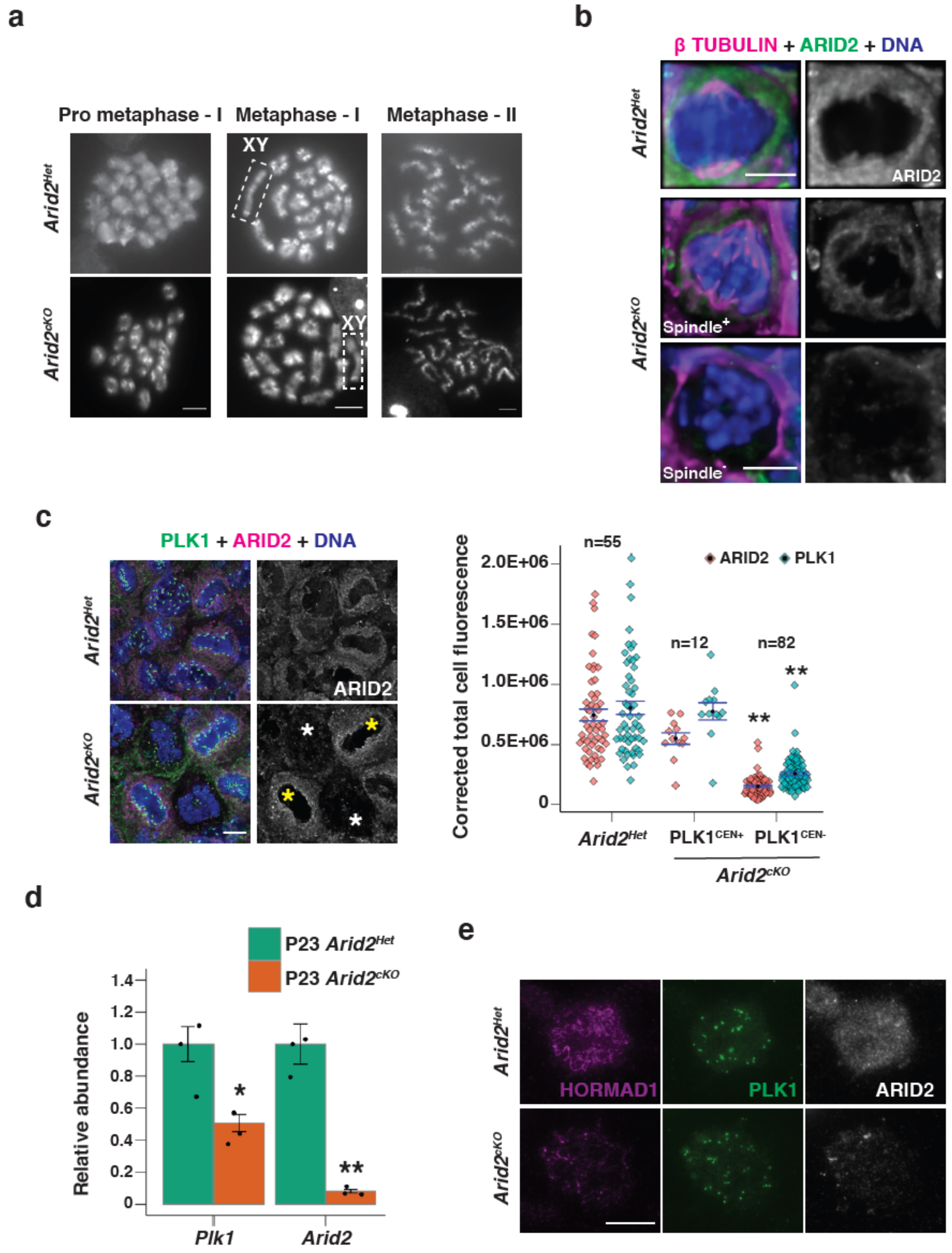

Supplementary Figure 2. Features of $Arid2^{cKO}$ spermatocytes. (a) DAPI-stained $Arid2^{Het}$ and $Arid2^{cKO}$ metaphase spreads. Representative images of metaphase spermatocytes observed in spreads prepared from P19, P23, P27 and 3-month $Arid2^{Het}$ and $Arid2^{cKO}$ testes. (b) Metaphase-I spermatocytes from $Arid2^{Het}$ and $Arid2^{cKO}$ testes cryosections immunolabelled for β-Tubulin (magenta) and ARID2 (green). Immunostainings were performed twice on testis cryosections obtained from two independent $Arid2^{Het}$ and $Arid2^{cKO}$ males. (c) PLK1 (green) and ARID2

(magenta) immunofluorescence (left) and quantification (right) of corrected total cell fluorescence (CTCF) from *Arid2*^{Het} and *Arid2*^{cKO} internal control (yellow asterisk, PLK1^{CEN+}) and mutant (white asterisk, PLK1 $^{CEN-}$) metaphase-I spermatocytes. ARID2 and PLK1 CTCF (y-axis) are expressed as mean (black dot) ± SEM (standard error of measurement). Number of spermatocytes quantified are indicated (n). (d) Quantitative RT-PCR analysis of *Plk1* and *Arid2* mRNA abundance (y-axis) in *Arid2*^{cKO} relative to *Arid2*^{Het} (control) spermatocyte enriched populations isolated at P23. mRNA abundance was normalized to reference gene, *Rplp0* and was expressed as mean ± SEM. Dots (black) represent values obtained from 3 biological replicates. (c,d) * $p = 0.0004$; ** $p < 0.0004$ were calculated by two-tailed unpaired Student's t-test. (e) *Arid2*^{Het} and *Arid2*^{cKO} diplotene spermatocytes immunolabeled for HORMAD1 (magenta), PLK1 (green) and ARID2 (white). (a-c, e) DNA counterstained with DAPI (blue). Scale bars: 5 µm, magnification: 100.8x. Immunostainings were performed twice on meiotic spreads obtained from juvenile males.

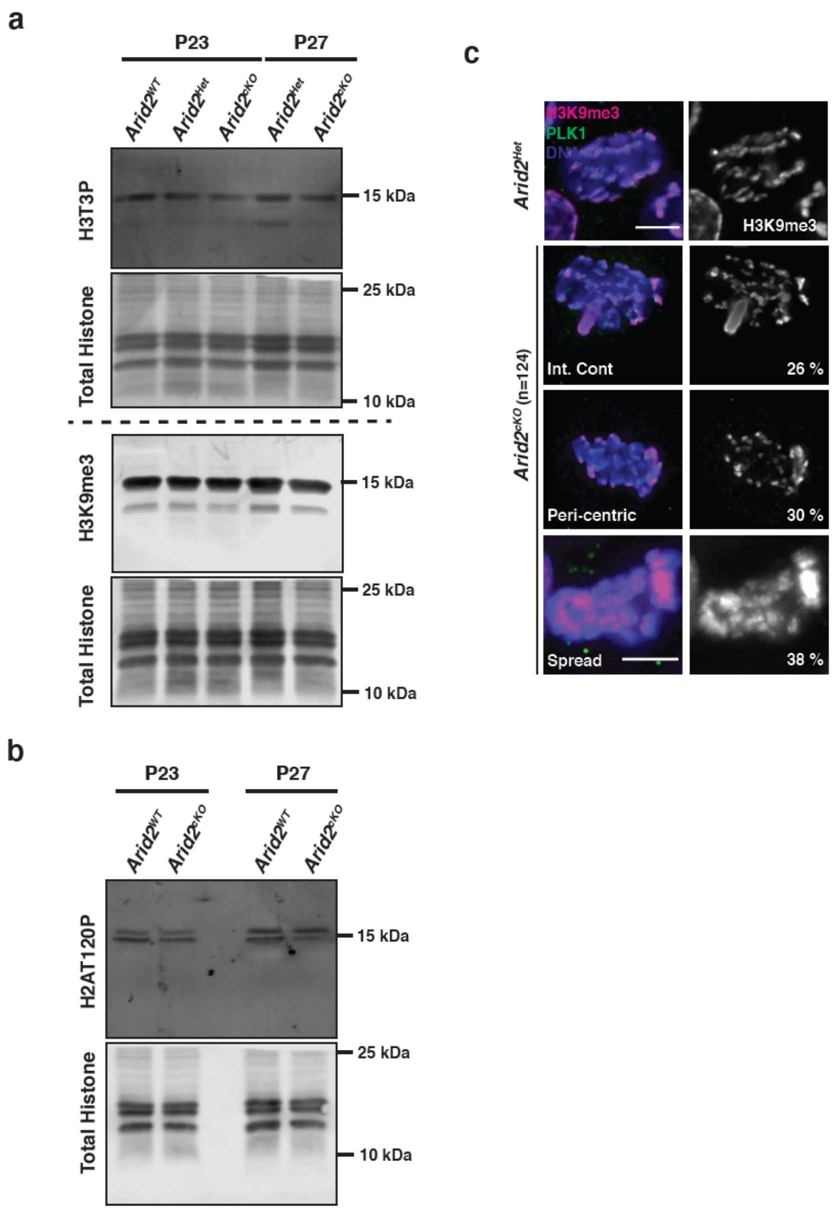

Supplementary Figure 3. Effect of ARID2 on abundance of centromeric and pericentric histone modifications. (a, b) Western blots on acid-extracted histones obtained from P23, P27 control and *Arid2*^{cKO} spermatogenic cells, showing (a) H3T3P (top), H3K9me3 (bottom), and (b) H2AT120P abundance. Total protein from each sample is displayed. Immunoblots were performed once each from P23 and P27 samples. (c) Control and *Arid2*^{cKO} metaphase-I spermatocytes immunolabelled for H3K9me2 (magenta) and PLK1 (green). DNA stained with DAPI. Total number (n) of metaphase-I spermatocytes scored from *Arid2*^{cKO} seminiferous tubules are indicated. Scale bar: 5 µm, magnification: 100.8x.

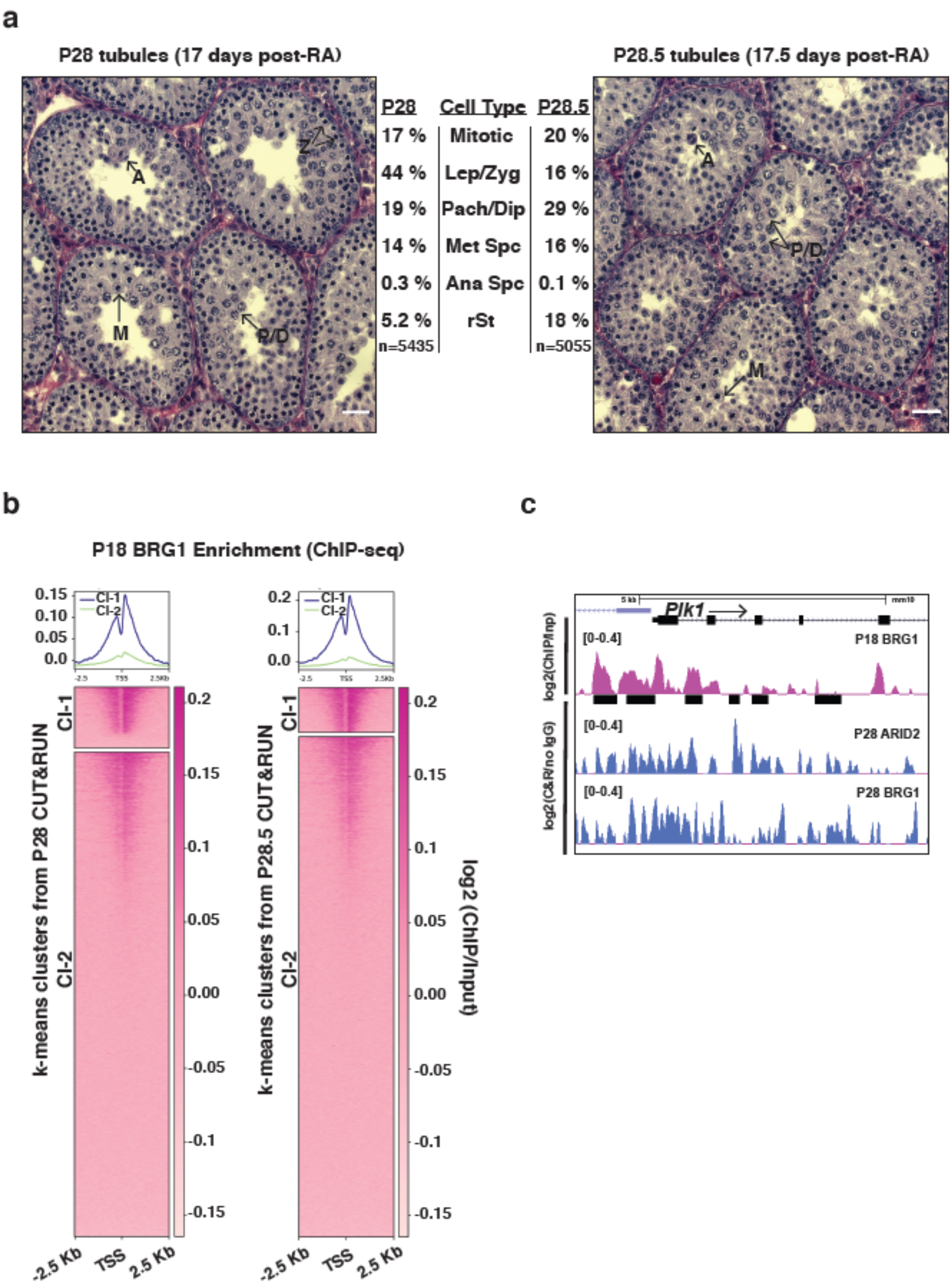

Supplementary Figure 4. ARID2 CUT&RUN from spermatogenic cells obtained after synchronization. (a) H&E-stained sections obtained from P28 and P28.5 synchronized testes. Proportion of cell types (%) determined from at least 30 tubules are indicated. n denotes total

cells scored. Lep: Leptotene, Zyg(Z): Zygotene, Pach(P): Pachytene, Dip(D): Diplotene, Met(M): Metaphase and Ana(A): Anaphase spermatocyte, RT: round spermatid. Scale bar: 100 µm, magnification: 20x. (b) Heatmap displaying BRG1 ChIP-seq enrichment from P18 testes. Enrichment is plotted over a 5Kb window centered at transcription start sites (TSS's) of genes associated with K-means clusters (Cl-1 and 2) identified at P28 and P28.5 from ARID2 CUT&RUN data. (c) Genome browser view of ARID2 and BRG1 enrichment at *Plk1*. Tracks representing P18 BRG1 ChIP-seq enrichment (magenta), P28 ARID2 and BRG1 CUT&RUN enrichment (blue) are displayed. Solid black bars denote P28 ARID2 CUT&RUN peak calls. Vertical viewing limits are indicated within parentheses.

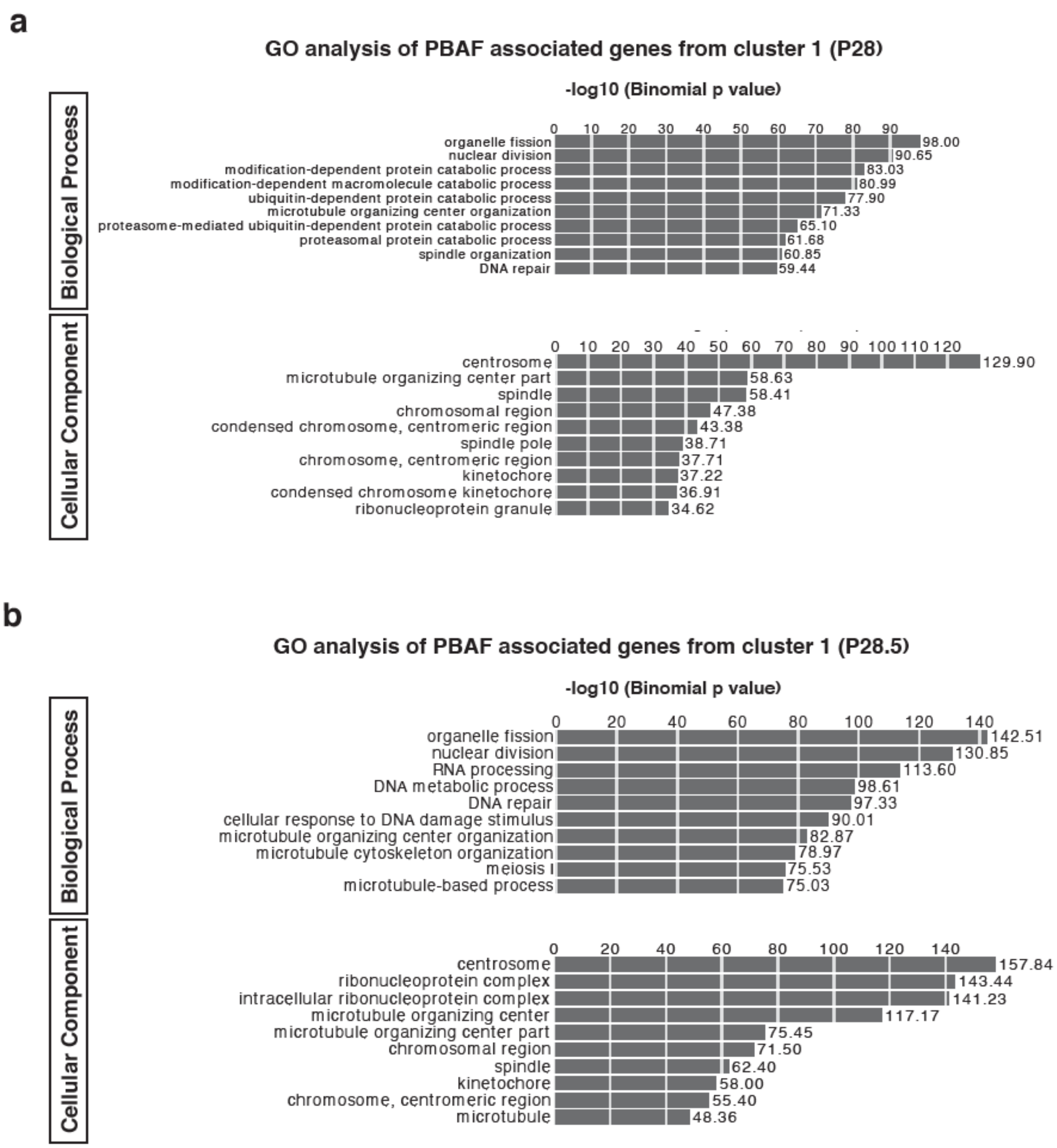

Supplementary Figure 5. Gene ontology (GO) analyses of PBAF associated genes. Biological process and cellular component GO terms associated with ARID2 target genes in k-means cluster 1 identified from (a) P28 and (b) P28.5 spermatocytes.

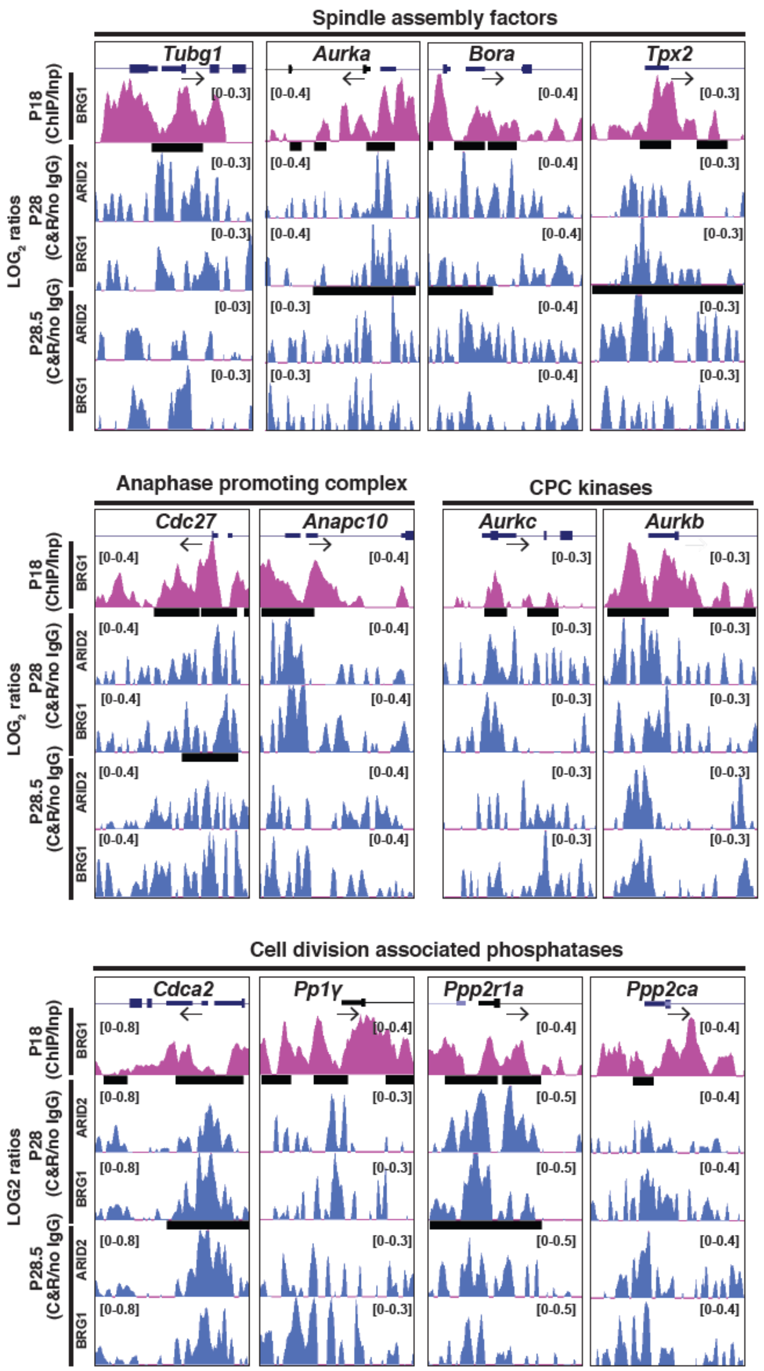

Supplementary Figure 6. Genome browser views of candidate PBAF associated genes. Tracks displaying BRG1 ChIP-seq enrichment from P18 testes (magenta), P28 and P28.5 ARID2/BRG1 CUT&RUN enrichment from synchronized testes (blue) at representative PBAF associated genes. Solid black bars denote P28 and P28.5 ARID2 CUT&RUN peak calls. Vertical viewing limits are indicated within parentheses.

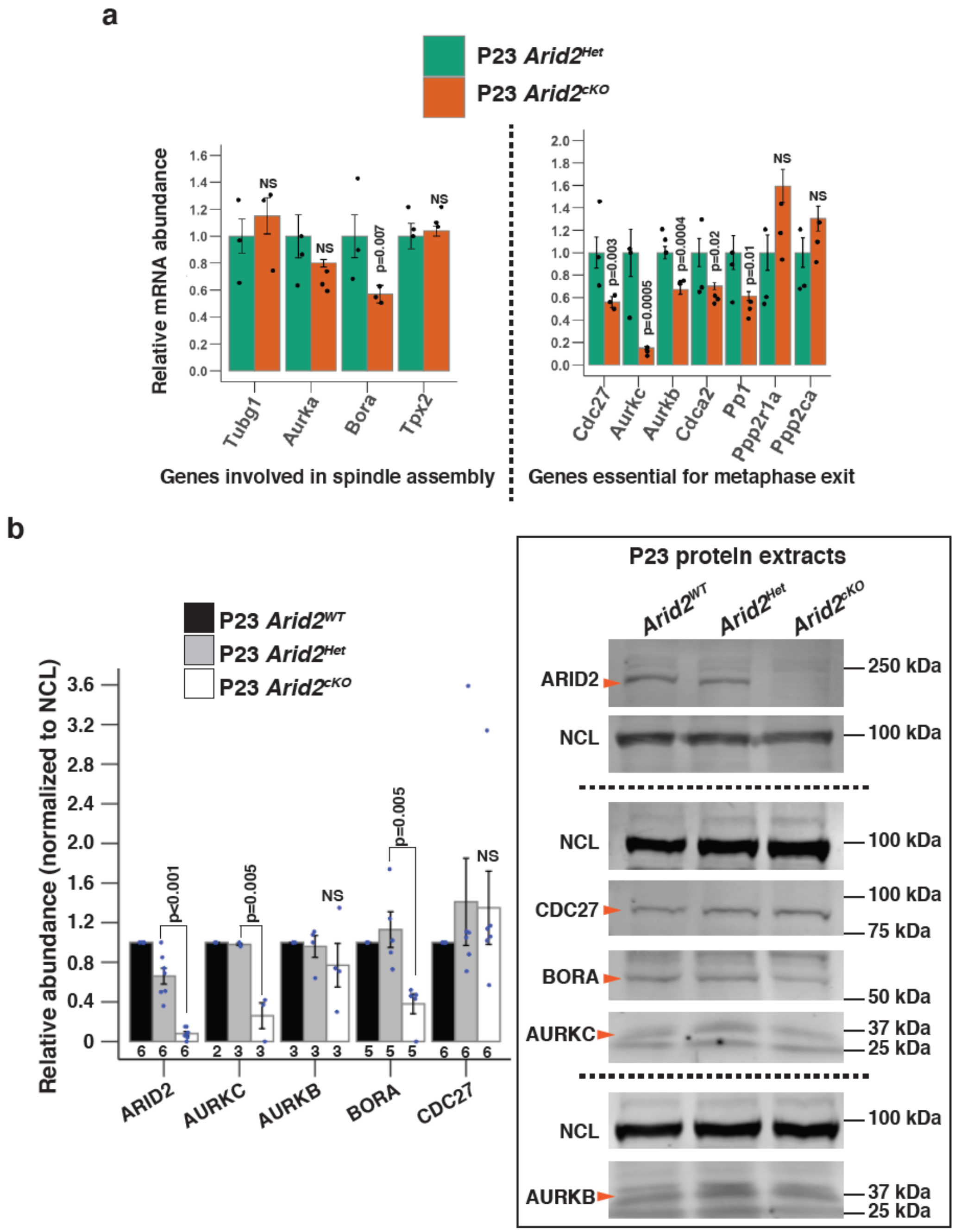

Supplementary Figure 7. PBAF regulates the expression of essential cell division genes. (a) Quantitative RT-PCR analysis of mRNA abundance of PBAF associated genes in *Arid2*^{cKO} relative to *Arid2*^{Het} (control) spermatocyte enriched populations isolated at P23. mRNA abundance was normalized to reference gene, *Rplp0*. Dots (black) represent values obtained from 3 biological replicates. (b) Western blot analysis of ARID2, AURKC, AURKB, BORA and CDC27 in

P23 *Arid2*^{WT}, *Arid2*^{Het} and *Arid2*^{cKO} spermatogenic cells. Protein levels were normalized to loading control, Nucleolin (NCL). Numbers below each bar indicate total independent measurements recorded (blue dots) for respective genotypes. Statistical testing of significance was performed on data from P23 *Arid2*^{Het} and *Arid2*^{cKO} replicates. (a,b) p-values calculated by two-tailed unpaired Student's t-test are indicated. NS: Not significant. Data are expressed as mean ± SEM.

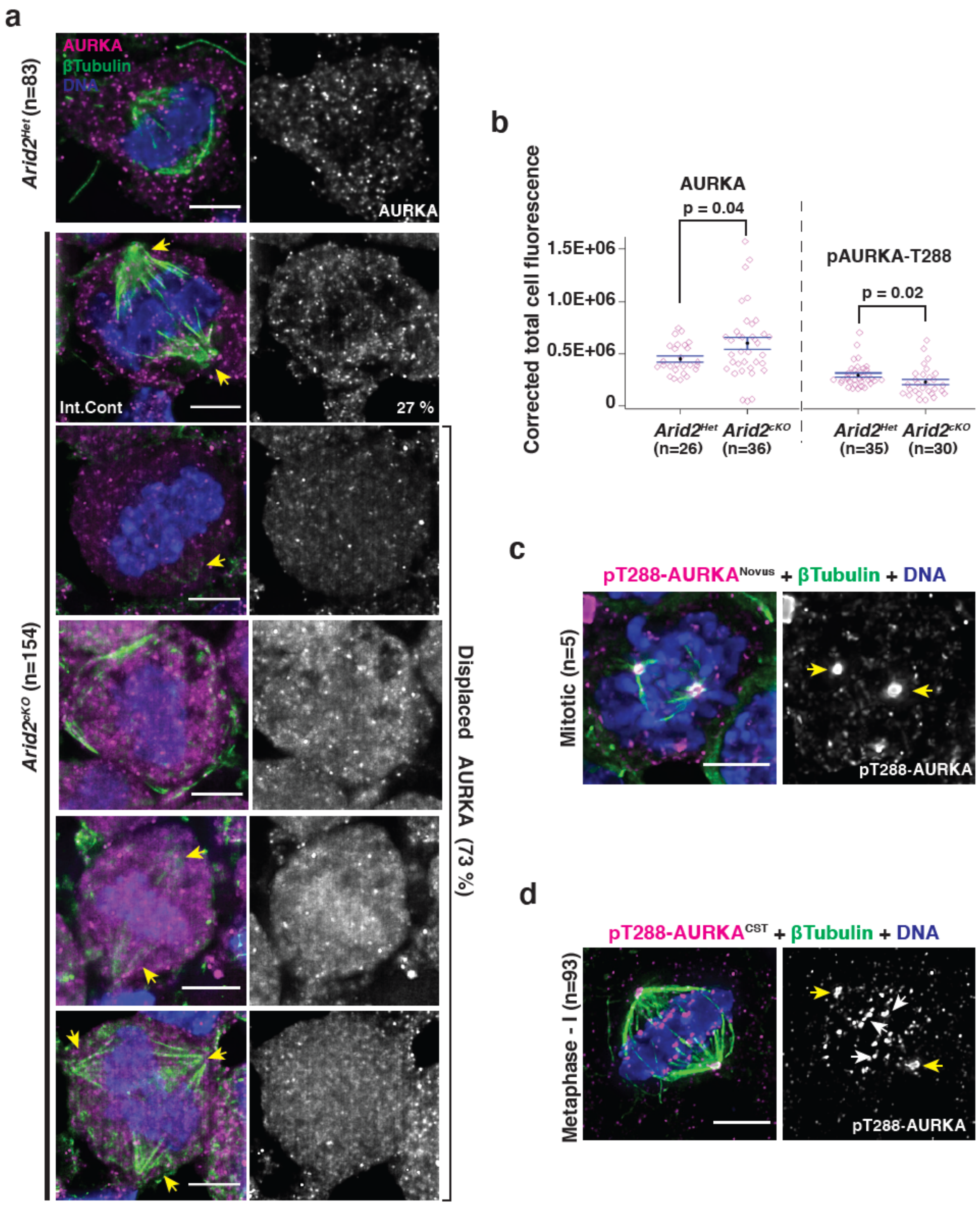

Supplementary Figure 8. ARID2 influences AURKA localization and abundance. (a) *Arid2^{Het}* and *Arid2^{cKO}* metaphase-I spermatocytes immunolabelled for AURKA (magenta) and β-Tubulin (green). DNA counterstained with DAPI (blue). Yellow arrowheads label spindle poles. Scale bars: 5 µm, magnification:100.8x. Number (n) of metaphase-I spermatocytes scored and proportion (%)

of internal controls (Int.Cont) and abnormal cells are indicated. (b) Quantification of AURKA and pT288-AURKA fluorescence from control and *Arid2*^{cKO} metaphase-I spermatocytes (n). Two-tailed unpaired Student's t-test p values are indicated. Data are expressed as mean (black dot) ± SEM. (c) Mitotic cell and (d) Metaphase-I spermatocyte immunolabelled for pT288-AURKA (magenta) and β-Tubulin (green). (c,d) DNA stained with DAPI. The identity of pT288-AURKA antibodies used are indicated (Novus and CST). Scale bar: 5 μm, magnification: 100x. Number (n) of cells observed are indicated.

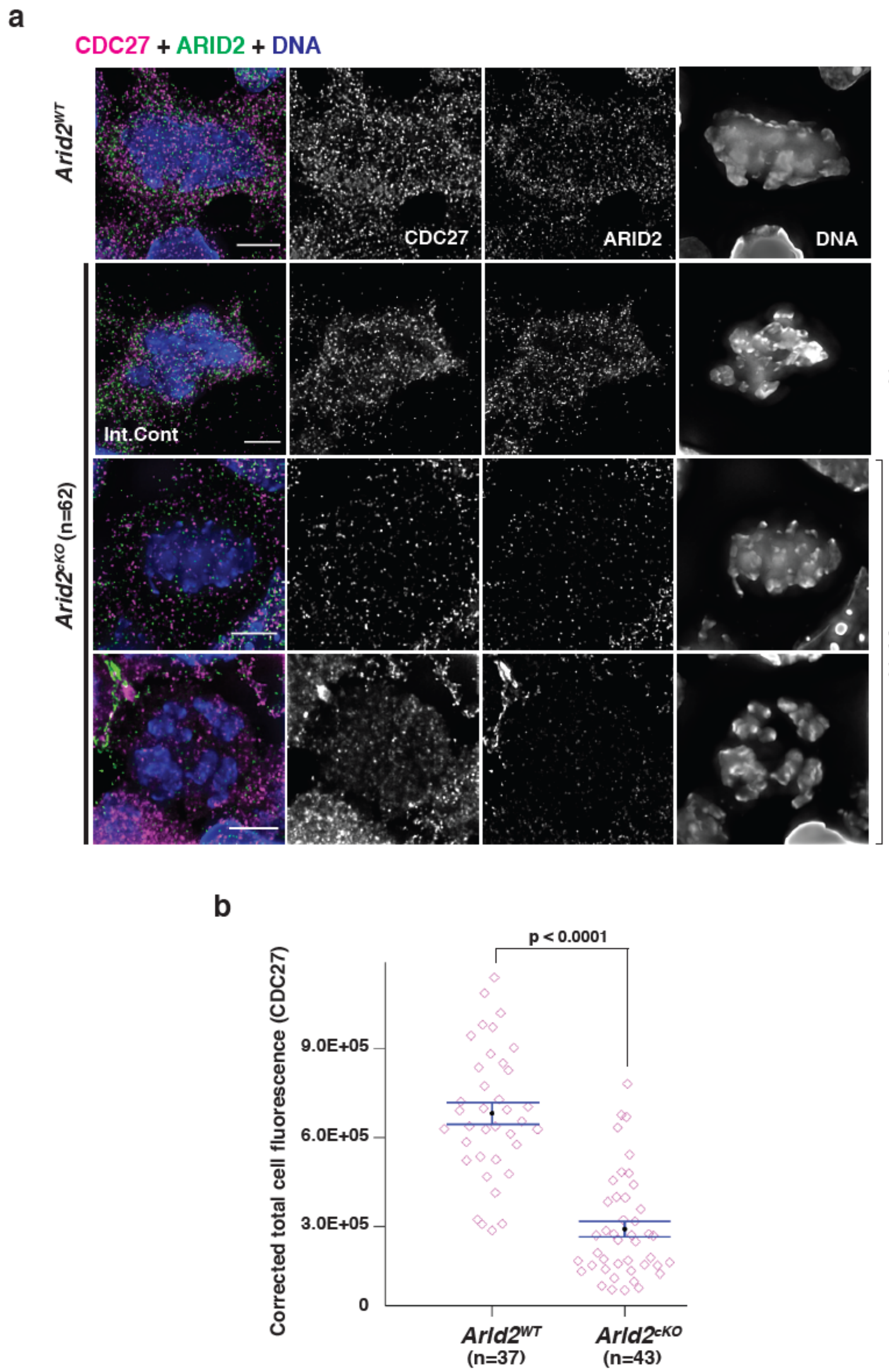

Supplementary Figure 9. ARID2 regulates CDC27 expression. (a) *Arid2*^{WT} and *Arid2*^{cKO} metaphase-I spermatocytes immunolabelled for CDC27 (magenta) and ARID2 (green). DNA counterstained with DAPI (blue). Scale bars: 5 µm, magnification:100x. Number (n) of metaphase-I spermatocytes scored and proportion (%) of internal controls (Int.Cont) and mutants are

indicated. (b) Quantification of CDC27 fluorescence from *Arid2*^{WT} and *Arid2*^{cKO} metaphase-I spermatocytes (n). Data are expressed as mean (black dot) ± SEM. Two-tailed unpaired Student's t-test p value is indicated.

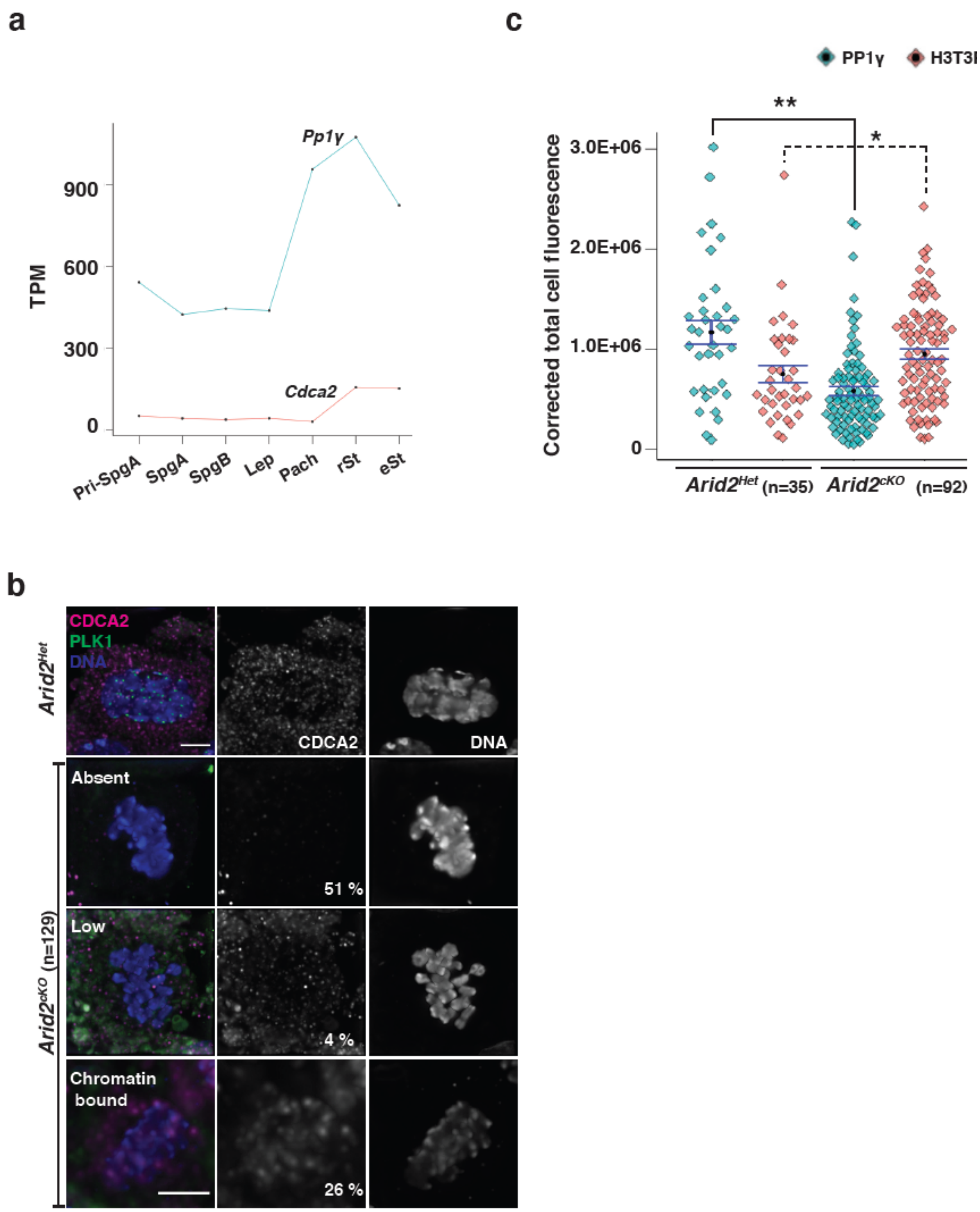

Supplementary Figure 10. ARID2 influences the abundance of CDCA2-PP1γ. (a) mRNA expression profile of *Cdca2* and *Pp1γ* in purified germ cell populations. TPM: Transcripts per million. (b) Control and *Arid2*^{cKO} metaphase-I spermatocyte squashes immunolabelled for PLK1 (green) and CDCA2 (magenta). DNA stained with DAPI. Scale bar: 5 µm, magnification: 100.8x. (c) Quantification of PP1γ and H3T3P fluorescence from control and *Arid2*^{cKO} metaphase-I spermatocytes (n). Data are expressed as mean (black dot) ± SEM. Two-tailed unpaired Student's t-test p value are indicated. ** $p < 0.0001$ and * $p = 0.05$.

Supplementary table 1. List of primary and secondary antibodies

Antibody	Vendor	Application
Rabbit anti-ARID2	Thermofisher (PA5-35857)	IF (1:100)<br>IP (10 µg)<br>WB (1:1000)<br>CUT&RUN (1:25)
Mouse anti- BRG1 (G-7)	SantaCruz Biotech (sc- 17796)	IF (1:100)<br>WB (1:1000)<br>CUT&RUN (1:25)
Rabbit anti- BRG1	Abcam (ab110641)	IP (7 µl)<br>IF (1:500)
Mouse anti- γH2Ax	Millipore (05-636)	IF (1:1000)
Rabbit anti NCL (Nucleolin)	Bethyl (A300-711A)	WB (1:2000)
Mouse anti- SYCP3	Abcam (ab97672)	IF (1:500)
Rabbit anti- γH2Ax	Cell signaling tech (9718)	IF (1:1000)
Rabbit anti- H3S10P	Millipore (06-570)	IF (1:500)
Mouse anti β-Tubulin	DHSB (E7)	IF (1:25)
Rabbit anti- CENPA	Abcam (ab33565)	IF (1:25)
Mouse anti γ-Tubulin	Proteintech (66320-1-Ig)	IF (1:100)
Mouse anti- PLK1[35-206]	Abcam (ab17056)	IF (1:200)<br>IP (9 µg)<br>WB (1:1000)
Guinea pig anti- HORMAD1	Gift from Dr. Atilla Töth, TU Dresden	IF (1:500)
Rabbit anti- H3T3P	Active Motif (39153)	IF (1:100)<br>WB (1:1000)
Rabbit anti-H3T3P	Cell signaling tech (13576S)	IF (1:2000)<br>ChIP (1.1µg)
Rabbit anti- H2AT120P	Active Motif (39392)	IF (1:100)<br>WB (1:1000)
Rabbit anti- H3K9me3	Abcam (ab8898)	IF (1:500)<br>WB (1:1000)
Rabbit anti-HK27me3	Cell signaling tech (9733)	ChIP (5 µg)
Rabbit anti- CDCA2	Sigma (HPA030049)	IF (1:100)
Rabbit anti-PPP1CC (PP1γ)	Proteintech (11082-1-AP)	Direct IF (1:50)
Rabbit anti- PPP2R1A	Proteintech (15882-1-AP)	IF (1:100)<br>IP (1.6 µg – 8 µg)<br>WB (1:800)
Mouse anti- PPP2CA	DHSB (CPTC-PP2A-4)	IF (1:10)<br>WB (1:20)
Rabbit anti – AURKA	Cell signaling tech (14475T)	IF (1:100)
Rabbit anti- pT288-AURKA	Novus biologicals (NB100-2371)	IF (1:100)
	Cell signaling tech (3079)	IF (1:100)
Rabbit anti- phosphoINCENP	Gift from Dr. Michael Lampson, University of Pennsylvania	IF (1:500)

Mouse anti – AURKC	Gift from Dr. Tang K. Tang, Institute of Biomedical Sciences, Academia Sinica, Taiwan, R.O.C.	IF (1:300)<br>WB (1:1250)
Mouse anti- AURKB	BD biosciences (611082)	IF (1:30)<br>WB (1:250)
Mouse anti- CDC27 (AF3.1)	Santa Cruz biotech (sc-9972)	IF (1:50)<br>WB (1:200)
Mouse anti- BORA (H-4)	Santa Cruz biotech (sc-393741)	IF (1:50)<br>WB (1:200)
**Secondary antibodies**		
Goat anti-mouse, Alexa fluor 488	ThermoFisher (A-11029)	IF (1:500)
Goat anti-mouse, Alexa fluor 568	ThermoFisher (A-11031)	IF (1:500)
Goat anti-rabbit, Alexa fluor 568	ThermoFisher (A-11036)	IF (1:500)
Goat anti- rabbit, Alexa fluor 488	ThermoFisher (A-11008)	IF (1:500)
Goat anti- guinea pig, Alexa fluor 568	ThermoFisher (A-11075)	IF (1:500)
Goat anti- rabbit, Alexa fluor 647	ThermoFisher (A-21245)	IF (1:500)
IRdye 680LT Goat anti-rabbit	LI-COR (926-68021)	WB (1:20,000)
IRdye 680LT Goat anti-mouse	LI-COR (925-68020)	WB (1:20,000)
IRdye 800CW Goat anti-mouse	LI-COR (926-32210)	WB (1:10,000)
AffiniPure Rabbit anti-Mouse IgG (H+L)	JacksonImmunoResearch (315-005-003)	CUT&RUN (1:100)
Guinea pig anti-Rabbit IgG (H+L)	Novus biologicals (NBP1-72763)	CUT&RUN (1:100)

Supplementary table 2. List of primers

RT-PCR primers		
Gene Symbol	Forward Primer (5' - 3')	Reverse Primer (5' - 3')
Tubg1	ATCTACCTGTCGGAGCATGG	CTGCCTCCCGATCTATGATG
Aurka	AGTTGGAAAGGGACATGGCT	CAGGCCTGGAGACACAGTTT
Bora	CAGCTGGCCATAATAAACCCT	GGCTTTTTGTCTTTTGTCTTCCA
Tpx2	CTTGATTGTGGCTTTGAGCG	CTTGTGACATTGCCCCAACT
Aurkc	GAGAAGGAGGGATTGGAGCA	AGTTGTACAGGCGAAGGATGTT
Aurkb	AGATTGCAGACTTTGGCTGG	AATCATCTCTGGGGGCAGAT
Cdca2	GAGGCAGACGGAGAGTCTAGG	AGGGAACCCAGACTGCTGAT
Pp1γ (Ppp1cc)	CTTCTCAGAGGGAACCACGA	TGTGAACGTTTTCCACAGCTT
Ppp2r1a	GGTTGAACGGACCAGAAGTG	TGTGAAGGTTCCCAGCTGTT
Ppp2ca	TGTGAAGGTTCCCAGCTGTT	TCTGCTCTCGTGATTCCCTC
Plk1	CCTTTGAGACCTCGTGCCTA	AGTGGGGTCTGTCTGAAGCA
Cdc27	CCTGAGGCACTGCAGAAAGT	CTGCGAGGAAAACTGCATCT
Rplp0	TTTGACAACGGCAGCATTTA	GTACCCGATCTGCAGACACAC
**ChIP-qPCR primers**		
Region/Gene	Forward Primer (5' - 3')	Reverse Primer (5' - 3')
Mini satellite	ACCACACTGTAGAACCTTTTAGA	ACACTGTTCTACAAATCCCGT
Major satellite	GGCAAGAAAACTGAAAATCATGGA	GTGTGCATTTCTCATTTTTCACG
Gdnf9	ACTTGCGGAGGATAGGTTGT	CTGTACAGAGCGAGGTAGGG
MMU19	GCTGGAAAGGTAAAGTGGCA	GGGGAAGAGAGAAAGGGACC
**Genotyping primers**		
Allele/transgene	Forward Primer (5' - 3')	Reverse Primer (5' - 3')
Arid2 fl/+	CTGCTTAGCCCAAAGGTGTC	GACAGTGACTTCAGCTGACC
Arid2 Δ	CTGCTTAGCCCAAAGGTGTC	CTGAGCCCAGGTGTTTTTGT
Stra8-Cre	GTGCAAGCTGAACAACAGGA	AGGGACACAGCATTGGAGTC
