# Supplementary material for: Mammalian SWI/SNF chromatin remodeler is essential for reductional meiosis in males
Source: Nat Commun. 2021 Nov 12;12:6581. doi: 10.1038/s41467-021-26828-1 (PMC8589837; doi:10.1038/s41467-021-26828-1)
Supplement: Supplementary file 3 — Reporting Summary [file 41467_2021_26828_MOESM3_ESM.pdf]

nature research

Corresponding author(s): Terry Magnuson

Last updated by author(s): Oct 5, 2021

Reporting Summary

Nature Research wishes to improve the reproducibility of the work that we publish. This form provides structure for consistency and transparency in reporting. For further information on Nature Research policies, see our Editorial Policies and the Editorial Policy Checklist.

Statistics

For all statistical analyses, confirm that the following items are present in the figure legend, table legend, main text, or Methods section.

n/a	Confirmed	
☐	☒	The exact sample size (n) for each experimental group/condition, given as a discrete number and unit of measurement
☐	☒	A statement on whether measurements were taken from distinct samples or whether the same sample was measured repeatedly
☐	☒	The statistical test(s) used AND whether they are one- or two-sided<br>*Only common tests should be described solely by name; describe more complex techniques in the Methods section.*
☒	☐	A description of all covariates tested
☒	☐	A description of any assumptions or corrections, such as tests of normality and adjustment for multiple comparisons
☐	☒	A full description of the statistical parameters including central tendency (e.g. means) or other basic estimates (e.g. regression coefficient) AND variation (e.g. standard deviation) or associated estimates of uncertainty (e.g. confidence intervals)
☐	☒	For null hypothesis testing, the test statistic (e.g. F, t, r) with confidence intervals, effect sizes, degrees of freedom and P value noted<br>*Give P values as exact values whenever suitable.*
☒	☐	For Bayesian analysis, information on the choice of priors and Markov chain Monte Carlo settings
☒	☐	For hierarchical and complex designs, identification of the appropriate level for tests and full reporting of outcomes
☒	☐	Estimates of effect sizes (e.g. Cohen's d, Pearson's r), indicating how they were calculated

Our web collection on statistics for biologists contains articles on many of the points above.

Software and code

Policy information about availability of computer code

Data collection	Leica LASX version 3.6.0.20104, Huygens essentials deconvolution software version 20.04, Zeiss Axiovision software version 4.8.1. For Next-gen sequencing, LI-COR Odyssey CLx imager, Image Studio Version 5.2.5, BIO-RAD real time PCR detection system CFX96, NOVASEQ 6000 S Prime flow cell (50 bp reads, paired end)
Data analysis	Fiji image analysis software (ImageJ) Version: 2.0.0-rc-69/1.52p, Bowtie2 version 2.4.1, samtools version 1.13, TrimGalore version 0.6.2, DeepTools version 3.2.0, WiggleTools version 1.2, Genomic Regions Enrichment of Annotation Tool (GREAT) version 4.0.4, SEACR version1.3, csaw version 1.4.1

For manuscripts utilizing custom algorithms or software that are central to the research but not yet described in published literature, software must be made available to editors and reviewers. We strongly encourage code deposition in a community repository (e.g. GitHub). See the Nature Research guidelines for submitting code & software for further information.

Data

Policy information about availability of data

All manuscripts must include a data availability statement. This statement should provide the following information, where applicable:

- Accession codes, unique identifiers, or web links for publicly available datasets
- A list of figures that have associated raw data
- A description of any restrictions on data availability

Data generated in this study are provided in supplementary information and source data. CUT&RUN data are deposited with GEO (Gene Expression Omnibus) under accession number GSE167539. RNA-seq data used in this study were previously published and are available from GEO under accession number GSE35005 . Published BRG1 ChIP-seq data is available from GEO under accession number GSE119179.

Field-specific reporting

Please select the one below that is the best fit for your research. If you are not sure, read the appropriate sections before making your selection.

[x] Life sciences   [ ] Behavioural & social sciences   [ ] Ecological, evolutionary & environmental sciences

For a reference copy of the document with all sections, see nature.com/documents/nr-reporting-summary-flat.pdf

Life sciences study design

All studies must disclose on these points even when the disclosure is negative.

Sample size

No sample size calculations were performed for any experiments.
For immunofluorescence studies, control and mutant cells were scored from testes cryosections and squashes obtained from multiple mice. We believe that the numbers of cells scored (>=50) for each immunofluorescence study were adequate given the scarce and transient nature of metaphase-I spermatocytes.

For western blots and Co-immunoprecipitations (Co-IP), samples sizes were chosen based on availability of control and mutant mice. Western blots were performed on testes protein extracts obtained from multiple juvenile (P19,P21,P23) and adult testes. Co-IPs were performed at least twice on testes protein lysates pooled from several wild type juvenile males. Reproducible results were obtained with selected sample sizes.

Quantitative RT-PCR was performed on 3 biological replicates from control and mutant animals to allow for statistical testing of significance.

ChIP was performed in triplicates while CUT&RUN was performed in duplicates. These sample sizes meet the ENCODE ChIP seq guidelines (https://dx.doi.org/10.1101%2Fgr.136184.111).

Data exclusions

No data were excluded

Replication

Spermatogenic phenotypes were determined from the comparative analysis of multiple independent mutant and litter mate controls. Metaphase-I arrest phenotype was reproducible across several Arid2cKO males. Histological staining's were reproducibly repeated from at least thrice separate testes and epididymal sections. Immunofluorescence data presented was confirmed using two separate preparations, cryosections and squashes. Moreover these studies were repeated at least twice to ensure reproducibility.
Each Co-immunoprecipition was reproducibly repeated twice. Moreover, reverse pull downs were incorporated in each trial to confirm interactions. Also, Co-IP with antibodies to two different subunits of the same complex (SWI/SNF) were performed to validate interactions.
Qualitative western blots were repeated reproducibly on at least two independent samples. For quantification of protein abundances in control and mutant samples, western blots were repeated a minimum of 3 and maximum of 6 times, depending on the availability of sample. Protein quantification data data were expressed as mean +/- SEM (standard error of measurement).
MNase sensitivity assays on control and mutant testes cryosections were performed over a range of MNase concentrations (0,20,200 Kunitz units), scoring >= 40 metaphase-I spermatocytes/section. Results were reproducible.

Randomization

Conditional knock-out animals and litter-mate controls were identified by genotyping . Testes samples were harvested from mutant and litter mate control mice at the same age. Apart from age no other covariate is anticipated

Blinding

Blinding was not performed for any experiments, as sample were collected from mice on the basis of their genotypes and developmental stage. In case of immunofluorescence data, examination of internal controls alongside mutant spermatocytes, obviated any risk of bias or misinterpretation of staining artefact's.

Reporting for specific materials, systems and methods

We require information from authors about some types of materials, experimental systems and methods used in many studies. Here, indicate whether each material, system or method listed is relevant to your study. If you are not sure if a list item applies to your research, read the appropriate section before selecting a response.

Materials & experimental systems

n/a	Involved in the study
☐	☒ Antibodies
☒	☐ Eukaryotic cell lines
☒	☐ Palaeontology and archaeology
☐	☒ Animals and other organisms
☒	☐ Human research participants
☒	☐ Clinical data
☒	☐ Dual use research of concern

Methods

n/a	Involved in the study
☐	☒ ChIP-seq
☒	☐ Flow cytometry
☒	☐ MRI-based neuroimaging

Antibodies

Antibodies used

List of primary antibodies (catalog #) and applications:
Rabbit anti-ARID2 Thermofisher (PA5-35857); IF (1:100),IP (10 μg),WB (1:1000),CUT&RUN (1:25)
Mouse anti- BRG1 (G-7) SantaCruz Biotech (sc- 17796); IF (1:100),WB (1:1000),CUT&RUN (1:25)
Rabbit anti- BRG1 Abcam (ab110641); IP (7 μl),IF (1:500)
Mouse anti- γH2Ax Millipore (05-636); IF (1:1000)
Rabbit anti NCL (Nucleolin) Bethyl (A300-711A); WB (1:2000)
Mouse anti- SYCP3 Abcam (ab97672); IF (1:500)
Rabbit anti- γH2Ax Cell signaling tech (9718); IF (1:1000)
Rabbit anti- H3S10P Millipore (06-570); IF (1:500)
Mouse anti β-Tubulin DHSB (E7); IF (1:25)
Rabbit anti- CENPA Abcam (ab33565); IF (1:25)
Mouse anti γ-Tubulin Proteintech (66320-1-Ig); IF (1:100)
Mouse anti- PLK1[35-206] Abcam (ab17056); IF (1:200),WB (1:1000), IP(9 μg)
Guinea pig anti- HORMAD1 Gift from Dr. Atilla Töth, TU Dresden IF; (1:500)
Rabbit anti- H3T3P Active Motif (39153); IF (1:100), WB (1:1000)
Rabbit anti-H3T3P Cell signaling tech (13576S); IF (1:2000),ChIP (1.1μg)
Rabbit anti- H2AT120P Active Motif (39392); IF (1:100),WB (1:1000)
Rabbit anti- H3K9me3 Abcam (ab8898); IF (1:500),WB (1:1000)
Rabbit anti-HK27me3 Cell signaling tech (9733); ChIP (5 μg)
Rabbit anti- CDCA2 Sigma (HPA030049); IF (1:100)
Rabbit anti-PPP1CC (PP1γ) Proteintech (11082-1-AP); Direct IF (1:50)
Rabbit anti- PPP2R1A Proteintech (15882-1-AP); IF (1:100), IP (1.6 μg – 8 μg), WB (1:800)
Mouse anti- PPP2CA DHSB (CPTC-PP2A-4); IF (1:10),WB (1:20)
Rabbit anti – AURKA Cell signaling tech (14475T); IF (1:100)
Rabbit anti- pT288-AURKA Novus biologicals (NB100-2371); IF (1:100)
Rabbit anti- pT288-AURKA Cell signaling tech (3079); IF (1:100)
Rabbit anti- phosphoINCENP Gift from Dr. Michael Lampson, University of Pennsylvania IF (1:500)
Mouse anti – AURKC Gift from Dr. Tang K. Tang, Institute of Biomedical Sciences, Academia Sinica, Taiwan, R.O.C. IF (1:300);WB (1:1250)
Mouse anti- AURKB BD biosciences (611082); IF (1:30), WB (1:250)
Mouse anti- CDC27 (AF3.1) Santa Cruz biotech (sc-9972); IF (1:50), WB (1:200)
Mouse anti- BORA (H-4) Santa Cruz biotech (sc-393741); IF (1:50), WB (1:200)

Secondary antibodies (catalog#) and dilutions used :
Goat anti-mouse, Alexa fluor 488 ThermoFisher (A-11029); IF (1:500)
Goat anti-mouse, Alexa fluor 568 ThermoFisher (A-11031); IF (1:500)
Goat anti-rabbit, Alexa fluor 568 ThermoFisher (A-11036); IF (1:500)
Goat anti- rabbit, Alexa fluor 488 ThermoFisher (A-11008); IF (1:500)
Goat anti- guinea pig, Alexa fluor 568 ThermoFisher (A-11075); IF (1:500)
Goat anti- rabbit, Alexa fluor 647 ThermoFisher (A-21245); IF (1:500)
IRdye 680LT Goat anti-rabbit LI-COR (926-68021); WB (1:20,000)
IRdye 680LT Goat anti-mouse LI-COR (925-68020); WB (1:20,000)
IRdye 800CW Goat anti-mouse LI-COR (926-32210); WB (1:10,000)
AffiniPure Rabbit anti-Mouse IgG (H+L) JacksonImmunoResearch (315-005-003); CUT&RUN (1:100)
Guinea pig anti-Rabbit IgG (H+L) Novus biologicals (NBP1-72763); CUT&RUN (1:100)

Validation

Rabbit anti-ARID2 Thermofisher (PA5-35857); Validated in this study using knockout tissue and validated by ThermoFisher https://www.thermofisher.com/antibody/product/ARID2-Antibody-Polyclonal/PA5-35857

Mouse anti- BRG1 (G-7) SantaCruz Biotech (sc- 17796); Knockout Validated in study by Menon et.al,2019, Development (doi:10.1242/dev.174094), and validated by SantaCruz Biotech, https://www.scbt.com/p/brg-1-antibody-g-7?requestFrom=search

Rabbit anti- BRG1 Abcam (ab110641); Knockout Validated in study by Menon et.al,2019, Development (doi:10.1242/dev.174094) and

validated by Abcam https://www.abcam.com/brg1-antibody-epncir111a-ab110641.html

Mouse anti- γH2Ax Millipore (05-636); Validated by Millipore sigma, https://www.emdmillipore.com/US/en/product/Anti-phospho-Histone-H2A.X-Ser139-Antibody-clone-JBW301,MM_NF-05-636

Rabbit anti NCL (Nucleolin) Bethyl (A300-711A); Validated by Bethyl labs, https://www.bethyl.com/product/A300-711A?referrer=search

Mouse anti- SYCP3 Abcam (ab97672); Validated by abcam, https://www.abcam.com/scp3-antibody-cor-10g117-ab97672.html

Rabbit anti- γH2Ax Cell signaling tech (9718); Validated by Cell signalling tech, https://www.cellsignal.com/products/primary-antibodies/phospho-histone-h2a-x-ser139-20e3-rabbit-mab/9718

Rabbit anti- H3S10P Millipore (06-570); Validated by Millipore sigma https://www.emdmillipore.com/US/en/product/Anti-phospho-Histone-H3-Ser10-Antibody-Mitosis-Marker,MM_NF-06-570

Mouse anti β-Tubulin DHSB (E7); Validated by Developmental studies hybridoma bank , https://dshb.biology.uiowa.edu/search?keywords=E7

Rabbit anti- CENPA Abcam (ab33565); Validated by abcam,https://www.abcam.com/cenpa-antibody-chip-grade-ab33565.html

Mouse anti γ-Tubulin Proteintech (66320-1-Ig); Validated by Proteintech, https://www.ptglab.com/products/tubulin-gamma-Antibody-66320-1-Ig.htm

Mouse anti- PLK1[35-206] Abcam (ab17056); Validated by abcam, https://www.abcam.com/plk1-antibody-35-206-ab17056.html

Guinea pig anti- HORMAD1; Knockout validated by Wojtasz L, et.al, 2009, PLOS Genetics (https://doi.org/10.1371/journal.pgen.1000702)

Rabbit anti- H3T3P Active Motif (39153); Validated by Active Motif, https://www.activemotif.com/catalog/details/39153/histone-h3-phospho-thr3-antibody-pab

Rabbit anti-H3T3P Cell signaling tech (13576S); Validated by CST, https://www.cellsignal.com/products/primary-antibodies/phospho-histone-h3-thr3-d5g1i-rabbit-mab/13576?site-search-type=Products&N=4294956287&Ntt=13576s&fromPage=plp&_requestid=622501

Rabbit anti- H2AT120P Active Motif (39392); Validated by Active Motif, https://www.activemotif.com/catalog/details/39391/histone-h2a-phospho-thr120-antibody-pab

Rabbit anti- H3K9me3 Abcam (ab8898); Validated by abcam, https://www.abcam.com/histone-h3-tri-methyl-k9-antibody-chip-grade-ab8898.html

Rabbit anti-HK27me3 Cell signaling tech (9733); Validated by Cell signalling tech, https://www.cellsignal.com/products/primary-antibodies/tri-methyl-histone-h3-lys27-c36b11-rabbit-mab/9733

Rabbit anti- CDCA2 Sigma (HPA030049); Validated by Sigma , https://www.sigmaaldrich.com/US/en/product/sigma/hpa030049?context=product

Rabbit anti-PPP1CC (PP1γ) Proteintech (11082-1-AP); Validated by Proteintech, https://www.ptglab.com/products/PPP1CC-Antibody-11082-1-AP.htm

Rabbit anti- PPP2R1A Proteintech (15882-1-AP); Validated by Proteintech, https://www.ptglab.com/products/PPP2R1A-Antibody-15882-1-AP.htm

Mouse anti- PPP2CA DHSB (CPTC-PP2A-4); Validated by Clinical Proteomics Technologies for Cancer https://antibodies.cancer.gov/detail/CPTC-PP2A-4#CPTC-PP2A-4-Characterization

Rabbit anti – AURKA Cell signaling tech (14475T); Validated by Cell signaling, https://www.cellsignal.com/products/primary-antibodies/aurora-a-d3e4q-rabbit-mab/14475?site-search-type=Products&N=4294956287&Ntt=14475t&fromPage=plp&_requestid=623650

Rabbit anti- pT288-AURKA Novus biologicals (NB100-2371); Validated by Novus biologicals, https://www.novusbio.com/products/aurora-a-antibody_nb100-2371

Rabbit anti- pT288-AURKA Cell signaling tech (3079); Validated by Cell signalling, https://www.cellsignal.com/products/primary-antibodies/phospho-aurora-a-thr288-c39d8-rabbit-mab/3079

Rabbit anti- phosphoINCENP; Validated by Salimian, K.J., et.al, 2011, Current Biology (https://doi.org/10.1016/j.cub.2011.06.015)

Mouse anti – AURKC ; Validated by Tang C.C., et.al, 2006, Developmental Biology (https://doi.org/10.1016/j.ydbio.2005.11.036) and

knockout validated by by Wellard, S.R., et.al, 2020, Journal of Cell Science (https://doi.org/10.1242/jcs.248831)

Mouse anti- AURKB BD biosciences (611082); Knockout validated by Wellard, S.R., et.al, 2020, Journal of Cell Science (https://doi.org/10.1242/jcs.248831) and validated by BD biosciences, https://www.bdbiosciences.com/en-us/products/reagents/microscopy-imaging-reagents/immunofluorescence-reagents/purified-mouse-anti-aim-1.611082

Mouse anti- CDC27 (AF3.1) Santa Cruz biotech (sc-9972); Validated by Santa Cruz biotech https://www.scbt.com/p/cdc27-antibody-af3-1?requestFrom=search

Mouse anti- BORA (H-4) Santa Cruz biotech (sc-393741); Validated by Santa Cruz biotech, https://www.scbt.com/p/bora-antibody-h-4?requestFrom=search

Animals and other organisms

Policy information about studies involving animals; ARRIVE guidelines recommended for reporting animal research

Laboratory animals	Arid2tm1c(EUCOMM)Wtsi and Stra8-cre mice were maintained on mixed genetic background. Wild type strains included male and female CD1 mice. Mutant and litter-mate control males were grown to 23 , 27, 37 days and 3 months of age before testes were harvested for data collection. Females of breeding age (2-5 months old) were used.
Wild animals	Study did not involve wild animals
Field-collected samples	Study did not involve field-collected samples
Ethics oversight	All animal work was carried out in accordance with approved IACUC protocols at the University of North Carolina at Chapel Hill

Note that full information on the approval of the study protocol must also be provided in the manuscript.

ChIP-seq

Data deposition

☒ Confirm that both raw and final processed data have been deposited in a public database such as GEO.

☒ Confirm that you have deposited or provided access to graph files (e.g. BED files) for the called peaks.

Data access links
May remain private before publication.

CUT&RUN data is submitted to GEO under accession number GSE167539.

Files in database submission

GSM5106940_ARID2d1-noIgG_CnRtrim.bw
GSM5106941_ARID2d2-noIgG_CnRtrim.bw
GSE167539_P28ARID2-noIgG_Avg.bw
GSM5106942_G7BRG1d1-noIgG_CnRtrim.bw
GSM5106943_abBRG1d1-noIgG_CnRtrim.bw
GSM5106945_ARID2m1-noIgG_CnRtrim.bw
GSM5106946_ARID2m2-noIgG_CnRtrim.bw
GSE167539_P28-5ARID2-noIgG_Avg.bw
GSM5106947_G7BRG1m1-noIgG_CnRtrim.bw
GSM5106948_abBRG1m1-noIgG_CnRtrim.bw
GSE167539_P28BRG1-noIgG_Avg.bw
GSE167539_P28-5BRG1-noIgG_Avg.bw
GSE167539_P28ARID2pks.bb
GSE167539_P28-5ARID2pks.bb

ARID2_d1_S1_L001_R1_001.fastq.gz
ARID2_d1_S1_L001_R2_001.fastq.gz
ARID2_d2_S2_L001_R1_001.fastq.gz
ARID2_d2_S2_L001_R2_001.fastq.gz
BRG1-G7_d1_S9_L001_R1_001.fastq.gz
BRG1-G7_d1_S9_L001_R2_001.fastq.gz
BRG1_d1_S3_L001_R1_001.fastq.gz
BRG1_d1_S3_L001_R2_001.fastq.gz
no_IgG_d1_S10_L001_R1_001.fastq.gz
no_IgG_d1_S10_L001_R2_001.fastq.gz
ARID2_m1_S11_L001_R1_001.fastq.gz
ARID2_m1_S11_L001_R2_001.fastq.gz
ARID2_m2_S12_L001_R1_001.fastq.gz
ARID2_m2_S12_L001_R2_001.fastq.gz
BRG1-G7_m1_S19_L001_R1_001.fastq.gz
BRG1-G7_m1_S19_L001_R2_001.fastq.gz
BRG1_m1_S13_L001_R1_001.fastq.gz

BRG1_m1_S13_L001_R2_001.fastq.gz
no_IgG_m1_S20_L001_R1_001.fastq.gz
no_IgG_m1_S20_L001_R2_001.fastq.gz

Genome browser session (e.g. UCSC)

https://genome.ucsc.edu/s/dmenon/mm10_PBAF_CUT&RUN

Methodology

Replicates	ARID2 and BRG1 CUT&RUN were performed in duplicates from P28 and P28.5 synchronized testes.<br>A no primary antibody control was included for CUT&RUN performed at each developmental stage.
Sequencing depth	CUT&RUN samples were sequenced on NOVASEQ 6000 S Prime flow cell in paired end 50 bp format.<br>Read summary for P28 samples:<br>ARID2_rep1: Total paired reads, 11,817,559 (11,142,934 mapped)<br>ARID2_rep2: Total paired reads, 23,103,783 (21,826,901 mapped)<br>BRG1_rep1: Total paired reads, 14,630,433 (13,807,309 mapped)<br>BRG1_rep2: Total paired reads, 12,757,676 (12,049,719 mapped)<br>no primary antibody control: Total paired reads, 18,234,415 (17,361,037 mapped)<br><br>Read summary for P28.5 samples:<br>ARID2_rep1: Total paired reads, 17,424,561 (16,377,133 mapped)<br>ARID2_rep2: Total paired reads, 25,607,258 (24,172,139 mapped)<br>BRG1_rep1: Total paired reads, 17,077,109 (16,075,243 mapped)<br>BRG1_rep2: Total paired reads, 17,446,764 (16,434,892 mapped)<br>no primary antibody control: Total paired reads, 16,130,747 (15,170,777 mapped)
Antibodies	Rabbit anti-ARID2; Thermofisher (PA5-35857)<br>Mouse anti- BRG1 (G-7); SantaCruz Biotech (sc- 17796)<br>Rabbit anti- BRG1; Abcam (ab110641)
Peak calling parameters	Read trimming with TrimGalore : trim_galore --trim-n --paired<br>bowtie2 alignment: bowtie2 --very-sensitive --no-mixed --dovetail --no-discordant --phred33 -I 10 -X 700<br><br>Transcription sites (TSSs) enriched for PBAF complex were were identified by performing k-means clustering on CUT&RUN signal with DeepTools plotHeatmap.<br><br>SEACR peaks were called relative to the control (no primary antibody) with "norm" and "relaxed" options
Data quality	PBAF genomic associations were identified based on ARID2 CUT&RUN data since it is a PBAF specific subunit<br><br>11553 TSSs were enriched for ARID2 relative to control samples (no primary antibody) at P28<br>8514 TSSs were enriched for ARID2 relative to control samples at P28.5<br><br>SEACR peaks:<br>892465 ARID2 peaks were called from P28 samples<br>2519 ARID2 peaks were called from P28.5 samples<br><br>87% of ARID2 enriched TSSs overlapped peaks called by SEACR for P28 samples<br>39% of ARID2 enriched TSSs overlapped peaks called by SEACR for P28.5 samples.
Software	Bowtie2 version 2.4.1, samtools version 1.13, TrimGalore version 0.6.2, DeepTools version 3.2.0, WiggleTools version 1.2, Genomic Regions Enrichment of Annotation Tool (GREAT) version 4.0.4, SEACR version1.3, csaw version 1.4.1
